# Supplementary material for: SyNDI: synchronous network data integration framework
Source: BMC Bioinformatics. 2018 Nov 6;19:403. doi: 10.1186/s12859-018-2426-5 (PMC6219086; doi:10.1186/s12859-018-2426-5)
Supplement: Supplementary file 5 — Files of the example presented in Additional file 4. This additional file is a zip package that contains all files used in Additional file 4. Detailed descriptions of each of these files are presented in a README file included in this zip package. (GZ 30411 kb) [file 12859_2018_2426_MOESM5_ESM.gz › SyNDITools/Meme2Fimo/meme_4.9.0_4.tar.gz!/meme_4.9.0/website/html/mast-intro.html]

MAST - Introduction


|  |  |
| --- | --- |
|  | MEME Suite The MEME Suite web application requires the use of JavaScript   Javascript doesn't seem to be available on your browser.  Introduction  MAST is a tool for searching biological sequence databases for sequences that contain one or more of a group of known motifs.  A motif is a sequence pattern that occurs repeatedly in a group of related protein or DNA sequences. Motifs are represented as position-dependent scoring matrices that describe the score of each possible letter at each position in the pattern. Individual motifs may not contain gaps. Patterns with variable-length gaps must be split into two or more separate motifs before being submitted as input to MAST.  MAST takes as input a file containing the descriptions of one or more motifs and searches a sequence database that you select for sequences that match the motifs. The motif file can be the output of the MEME motif discovery tool or any file in the appropriate format.  MAST outputs details on the top scoring sequences ordered by score. The score is calculated as the strength of the combined match of the sequence to all motifs. The basic details shown include the sequence name, *E*-value indicating the match strength and the strong motif occurrences shown in the form of a block diagram. Expanded details available include the sequence comment, combined *p*-value and annotated sequence.  MAST works by calculating match scores for each sequence in the database compared with each of the motifs in the group of motifs you provide. For each sequence, the match scores are converted into various types of p-values and these are used to determine the overall match of the sequence to the group of motifs and the probable order and spacing of occurrences of the motifs in the sequence. |
